# Shedding light on ovothiol biosynthesis in marine metazoans

**DOI:** 10.1038/srep21506

**Published:** 2016-02-26

**Authors:** Immacolata Castellano, Oriana Migliaccio, Salvatore D’Aniello, Antonello Merlino, Alessandra Napolitano, Anna Palumbo

**Affiliations:** 1 Department of Biology and Evolution of Marine Organisms, Stazione Zoologica Anton Dohrn, Naples, Italy; 2 Department of Chemical Sciences, University of Naples “Federico II”, Italy

## Abstract

Ovothiol, isolated from marine invertebrate eggs, is considered one of the most powerful antioxidant with potential for drug development. However, its biological functions in marine organisms still represent a matter of debate. In sea urchins, the most accepted view is that ovothiol protects the eggs by the high oxidative burst at fertilization. In this work we address the role of ovothiol during sea urchin development to give new insights on ovothiol biosynthesis in metazoans. The gene involved in ovothiol biosynthesis *OvoA* was identified in *Paracentrotus lividus* genome (*PlOvoA*). *PlOvoA* embryo expression significantly increased at the pluteus stage and was up-regulated by metals at concentrations mimicking polluted sea-water and by cyclic toxic algal blooms, leading to ovothiol biosynthesis. *In silico* analyses of the *PlOvoA* upstream region revealed metal and stress responsive elements. Structural protein models highlighted conserved active site residues likely responsible for ovothiol biosynthesis. Phylogenetic analyses indicated that OvoA evolved in most marine metazoans and was lost in bony vertebrates during the transition from the aquatic to terrestrial environment. These results highlight the crucial role of OvoA in protecting embryos released in seawater from environmental cues, thus allowing the survival under different conditions.

Exposure to metals, toxins and more generally pollutants induces oxidative stress in marine organisms, which are able to respond with the induction of both enzymatic and non-enzymatic antioxidant defenses. These systems are necessary for sustaining marine life by maintaining a fine intracellular redox balance and minimizing undesirable cellular damage caused by reactive oxygen species (ROS). In addition to the well-known antioxidant enzymes, superoxide dismutase, catalase, and several peroxidases, organisms produce low molecular-weight non-enzymatic antioxidants, which also function as direct ROS scavengers^1^. Besides the ubiquitous tripeptide glutathione containing the reactive cysteine^2^, some marine organisms produce ovothiol, the π-methyl-5-thiohistidine, first isolated from ovary, eggs and biological fluids of sea urchins and cephalopods^3^,^4^,^5^. Ovothiol exists in three forms (ovothiol A, B and C) differing in the degree of methylation at the aminoacidic side chain. In particular, ovothiol A is unmethylated, whereas ovothiol B and C are mono- or di-methylated, respectively. The sea urchin *Paracentrotus lividus* eggs contain high concentrations (millimolar) of ovothiol A, whereas *Strongylocentrotus purpuratus* eggs contain high concentrations of ovothiol C. Thanks to the aromaticity of the imidazole ring, ovothiol possesses a very acidic thiol group (pKa = 1.4), when compared to the other cellular thiols, such as glutathione, trypanothione or ergothioneine^6^,^7^,^8^. This unusual chemical feature confers ovothiol a thiolate anion form over a wide range of pH values^9^ and makes it an efficient scavenger of radicals and peroxides^10^,^11^ by providing also protection against peroxynitrite-induced damage^12^. These *in vitro* studies suggest that ovothiol is involved in the balancing of cellular redox homeostasis. It was previously hypothesized that, in sea urchin eggs, the reduced form of ovothiol is oxidized by hydrogen peroxide, produced during the oxidative burst at fertilization and is then regenerated by intracellular glutathione, thus providing a non-enzymatic glutathione peroxidase-like activity^13^,^14^,^15^. This system seems more efficient than catalase in destroying hydrogen peroxide at the concentrations produced during fertilization. The occurrence of ovothiol in other organisms and its involvement in different biological processes have also been described. In some pathogens, especially trypanosomes such as *Crithidia fasciculata* and *Leishmania donovani,* ovothiol has been suggested to be involved in the protection of the parasites from oxidative stress produced by macrophages during infection^16^,^17^. In the halotolerant green alga *Dunaliella salina* ovothiol has been proposed as a redox regulator in chloroplasts^18^, whereas in the marine polychaete *Platynereis dumerilii* it has been suggested to act as a male pheromone during coupling^19^. In other marine organisms, ovothiol was identified in substructures of larger natural products, whose functions are still unknown. For example, ovothiol is a component of adenochrome, the iron-binding protein in *Octopus vulgaris*^20^,^21^, and of imbricatine, an alkaloid produced by the sea star *Dermasterias imbricate*^22^.

Thanks to its chemical features, ovothiol has been receiving increasing interest for its potential therapeutic use in humans. Some ovothiol analogues were synthetized and their antioxidant properties studied in *in vitro* experiments^23^. Among these, 1-methyl-2-(3-trifluoromethylphenyl)-4-mercaptoimidazole has been shown to be a potent agent in mammalian cerebral protection^24^. Moreover, ovothiol A isolated from sea urchin eggs has been recently reported to induce autophagy in liver carcinoma cell lines, suggesting a potential role in regulating cancer cell growth^25^. In addition, the enzyme which catalyzes the first step of ovothiol biosynthesis, 5‐histidylcysteine sulfoxide synthase (OvoA), recently characterized from the bacterium *Erwinia tasmaniensis* and the pathogenic protist *Trypanosoma cruzi*^26^, has been proposed as a target of anti-infective therapy. This enzyme is an iron (II) dependent sulfoxide synthase, which catalyzes the S-conjunction of cysteine with histidine in the presence of molecular oxygen and *S*-adenosyl methionine. Subsequently, a sulfoxide lyase cleaves the sulfur carbon bond in the cysteine residue leaving 5-thiohistidine, finally methylated at the imidazole ring^27^,^28^,^29^. OvoA enzyme is the homologous of an iron dependent sulfoxide synthase involved in ergothioneine biosynthesis (EgtB), a 2-thiohistidine, produced in some actinobacteria, cyanobacteria, and fungi^30^. The key step in the biosynthesis of these thiohistidines is the oxidative insertion of a sulfur atom into the C2 or C5 position of the imidazole ring of L-histidine. This represents an unusual and completely novel reaction with respect to known mechanisms for enzymatic C-S bond formation^31^. *In silico* analysis has revealed the presence of OvoA mainly in bacteria, protists, fungi, and some animals^26^.

The aim of the present work is to address the biological role of OvoA in marine metazoans. To this aim, sea urchins represent exceptional models for evolutionary, ecotoxicological, and developmental biology studies. We have identified and analyzed the nucleotide sequence of the gene locus for *OvoA* in the sea urchin *P. lividus* in comparison with the orthologous one in *S. purpuratus*. We have investigated the role of OvoA in mediating *P. lividus* embryos response to environmental stress factors, including heavy metals and natural algal blooms. Evolutionary analysis of OvoA in metazoans has allowed us to hypothesize the involvement of ovothiol in protecting eggs and embryos released in the seawater column from environmental cues.

## Results

### *PlOvoA* and *SpOvoA* gene loci structure

**Figure 1:**
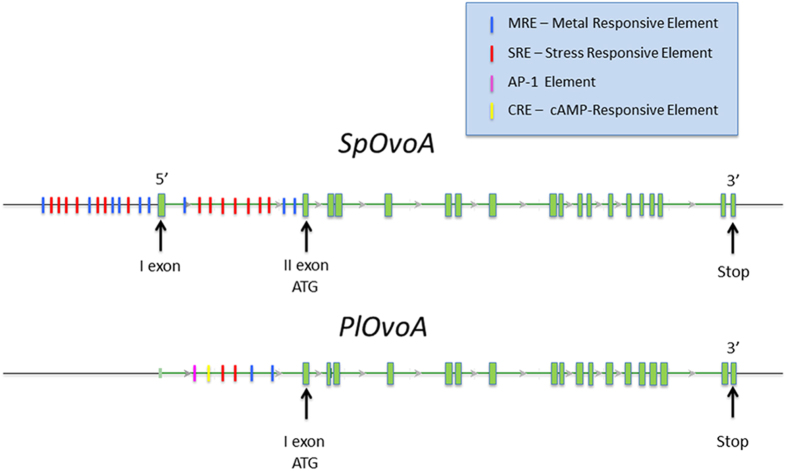
Schematic representation of *SpOvoA* and *PlOvoA* gene loci. The exons are illustrated as green boxes. The promoter responsive elements are reported in different colors as indicated in the figure legend.

The *OvoA* genes in the sea urchins *P. lividus* and *S. purpuratu*s genomes were identified by tblastx using as sequence query the previously characterized OvoA of *E. tasmaniensis* and *T. cruzi. SpOvoA* is a multi-exonic gene with 19 exons. *Pl*OvoA shows a similar gene structure (Fig. 1). The *in silico* analysis of upstream regions of these genes revealed the presence of Metal Responsive Element (MRE) and Stress Responsive Element (SRE). In the 12 kb upstream region of *Sp*OvoA, including the first intron, 9 putative MREs and 14 putative SREs were identified (Fig. 1). In *PlOvoA* orthologous region, besides 2 MREs and 2 SREs, 1 activator protein 1 (AP-1) and 1 cAMP-Responsive Element (CRE) were found. However, low quality of the assembled sequences of *P. lividus* genome in that area did not allow to rule out the presence of other responsive *cis*-elements.

### *PlOvoA* expression profile during embryo development and response to environmental factors

**Figure 2:**
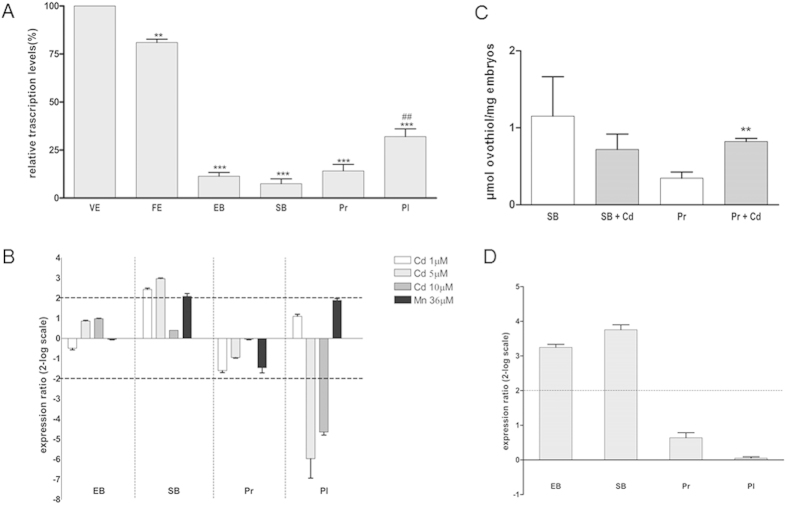
*PlOvoA* expression profile and response to environmental stress conditions. (**A**) *PlOvoA* expression during embryo development. Eggs and embryos collected at different development stages (virgin eggs VE, fertilized eggs FE, early blastula EB, swimming blastula SB, prisma Pr and pluteus Pl) were examined for gene expression by Real Time qPCR. Fold differences in the expression levels of *PlOvoA* with respect to the reference gene *Pl-Z12-1* were expressed in percentage with respect to highest levels of mRNA in VE (100%). *represents the significance respect to VE, **P < 0.01, ***P < 0.001. #represents the significance respect to Pr, ^##^*P* < 0.01. **(B**) *Pl OvoA* expression analysis in developing embryos after metal treatment. FE were treated with 1, 5, 10 μM Cd^2+^ and 36 μM Mn^2+^ and different developmental stages were examined for the transcriptional expression of *PlOvoA*. Data are reported as a fold difference in *PlOvoA* expression levels, compared to control (mean ± SEM), embryos developed in seawater without metals. Fold differences greater than ± 2 (see dotted horizontal guidelines at values of 2 and −2) were considered significant. (**C**) Ovothiol levels in developing embryos after metal treatment. FE were treated with 5 μM Cd^2+^ and SB and Pr were examined for ovothiol production. Data are reported as μmol ovothiol/mg of embryos. Results are representative of 3 independent experiments and expressed as means ± SEM, and analyzed by unpaired t-test. *represents the significance respect to the control, **P < 0.01. **D**. *Pl*OvoA expression analysis in developing embryos after maternal exposure to *O.* cf *ovata* bloom. Sea urchin females, exposed to *Ostreopsis* cf. *ovata* bloom in July at Gaiola site, were collected in October and fertilized. Different developmental stages (EB, SB, Pr and Pl) were examined for *PlOvoA* expression. Data (mean ± SEM) are reported as a fold difference in *PlOvoA* expression levels, compared to control embryos derived from sea urchins collected at control site. Fold differences greater than ± 2 were considered significant.

Initial experiments were performed to examine by quantitative RT-PCR *PlOvoA* transcription during *P. lividus* development from virgin eggs to pluteus stage larvae (Fig. 2A), using the zinc-finger transcription factor *Pl-Z12-1* as reference gene, whose expression remained constant in all developmental stages. Immediately after fertilization, *PlOvoA* expression was slightly reduced compared to the highest value in the unfertilized eggs and then strongly decreased at the early and the swimming blastula stages. Thereafter, *PlOvoA* expression levels significantly increased at the pluteus stage.

In subsequent experiments, sea urchin fertilized eggs were treated with Cd^2+^ and Mn^2+^ at concentrations mimicking polluted seawater and previously shown to affect development^32^. *PlOvo*A expression was followed during embryo development until the pluteus stage with respect to untreated embryos (Fig. 2B). A significant increase of *PlOvoA* mRNA was observed at the swimming blastula stage after exposure to 1 and 5 μM Cd^2+^, whereas at the early blastula and prism stages, the gene transcription was unaffected. On the contrary, at the pluteus stage *PlOvoA* mRNA was strongly down-regulated after exposure to 5 and 10 μM Cd^2+^. In the presence of Mn^2+^, the relative expression ratio of *PlOvoA* at the swimming blastula stage was close to the minimum value (2) considered significant. No appreciable gene regulation was observed in other developmental stages.

To understand if the metal-induced up-regulation of *PlOvoA* gene reflected also an increase in ovothiol biosynthesis, the amount of the thiohistidine was determined by HPLC analysis at the swimming blastula stage, when *PlOvoA* transcription increased, and at the subsequent prism stage, after exposure of fertilized eggs to the highest effective concentration of Cd^2+^ (5 μM). Ovothiol levels significantly increased at the prism stage after exposure to the metal, with respect to the control, whereas no significant increase was detected at the swimming blastula stage (Fig. 2C).

In order to assess *PlOvoA* gene regulation by other environmental stress factors, we examined its expression in the offspring of sea urchins cyclically exposed to a natural toxic bloom of the dinoflagellate *Ostreopsis* cf. *ovata* occurred at the Gaiola Marine Protected Area in the Gulf of Naples. Females were collected in October during the reproductive season, after *O.* cf *ovata* bloom, which occurred in July. The eggs were fertilized in the laboratory and the offspring analyzed for mRNA expression of the *PlOvoA* gene at the different developmental stages. A significant increase of *PlOvoA* mRNA was observed at the early and swimming blastula stages with respect to embryos derived from females harvested at the control site, Castel dell’Ovo, which is known to harbor *O.* cf. *ovata* at negligible concentrations (Fig. 2D).

### Identification and characterization of *Pl*OvoA protein

**Figure 3:**
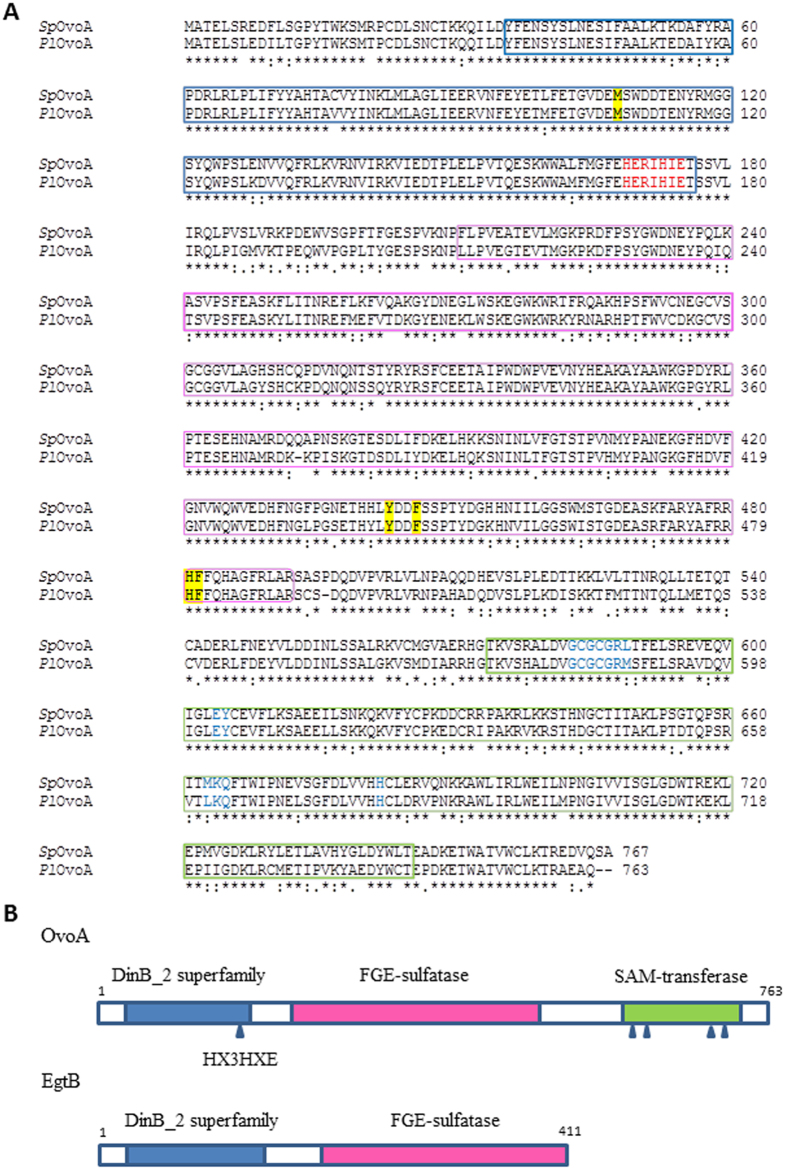
Characterization of OvoA protein in sea urchins. (**A**) Sequence alignment of *Pl*OvoA and *Sp*OvoA. DinB superfamily domain (36–176 aa, *Pl*OvoA) in the N-terminal region is boxed in blue. The putative iron binding motif (HX3HXE) is indicated in red. The FGE-sulfatase domain (211–491 aa) is boxed in magenta and the SAM-transferase domain (572–743) in the C-terminal region is boxed in green. The residues (581–587, 602–603, 661–663, 680) belonging to the SAM-binding site are indicated in blue. The putative residues accounting for binding to cysteine and histidine are highlighted in yellow. (**B**) Schematic representation of OvoA and EgtB primary structure. DinB_2 superfamily domain is boxed in blue and the putative iron-binding site is indicated by an arrow, FGE-sulfatase domain is boxed in magenta. SAM-transferase domain is boxed in green. SAM binding sites are highlighted by arrows.

The open reading frames of *Pl*OvoA and *Sp*OvoA code for a protein of 763 and 767 amino acids (aa), respectively. The two proteins are highly similar (83% of sequence identity) and share 34–35% of sequence identity with *E. tasmaniensis* OvoA. Both protein primary structures contain a DinB superfamily domain (36–176 aa, *Pl*OvoA) and a Formylglycine-generating sulfatase (FGE-sulfatase) domain (211–491 aa) in the N-terminal region and a S-adenosylmethionine -methyltransferase (SAM-transferase) domain (572–743) in the C-terminal region (Fig. 3A). The DinB superfamily domain contains the conserved HX3HXE putative iron-binding motif. Moreover, the residues (581–587, 602–603, 661–663, 680) considered to be involved in the formation of SAM-binding site are conserved with the bacterial orthologous gene. OvoA shares two protein domains with EgtB, the DinB superfamily domain and the FGE-sulfatase domain but differs for the additional C-terminal putative SAM-transferase domain^26^ (Fig. 3B).

**Figure 4:**
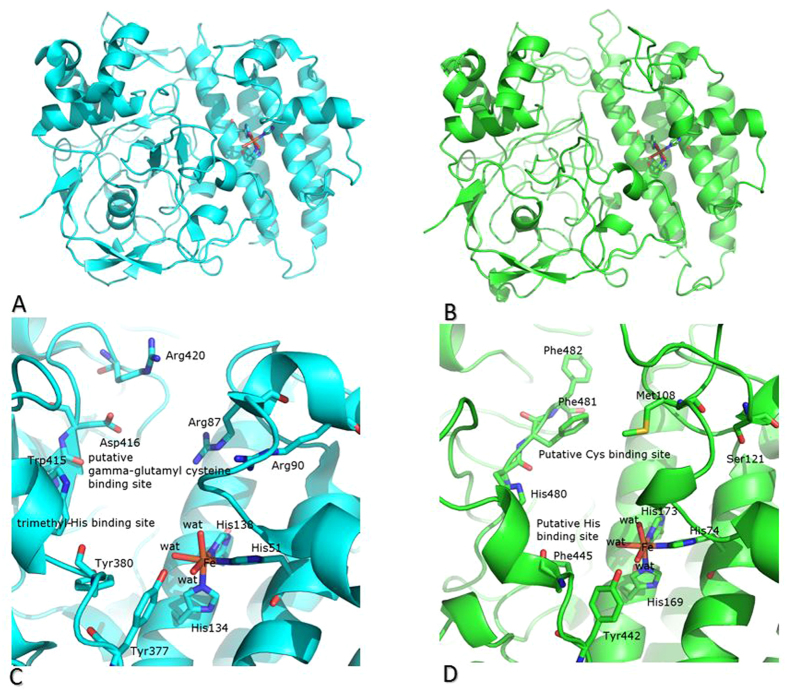
Structural model of *Pl*OvoA. (**A,B**) Ribbon representation of the two domains shared by *Mt*EgtB (panel A, in cyan) and *Pl*OvoA (panel B, in green). The iron-binding site and the conserved His residues are also shown as ball and stick. (**C,D**) Comparison between the active sites of *Mt*EgtB and *Pl*OvoA. *Mt*EgtB (cyan) and *Pl*OvoA (green) catalyze C-S bond formation and sulfoxidation between gamma-glutamyl cysteine and N-alpha-trimethyl histidine or between cysteine and histidine as the central steps in the synthesis of ergothioneine and ovothiol, respectively. Residues involved in the recognition of iron are conserved, whereas those involved in the recognition of N-alpha-trimethyl histidine or important for the binding of gamma-glutamyl cysteine in *Mt*EgtB are not conserved in *Pl*OvoA, according to the different substrates (N-alpha-trimethyl histidine versus His and gamma-glutamyl cysteine versus cysteine) of these enzymes.

The structure of *Pl*OvoA, excluding the C-terminal additional domain, has been modeled and compared with that of EtgB from *Mycobacterium thermoresistibile* (*Mt*EgtB, 33% sequence similarity). As expected, the overall structure of *Pl*OvoA is similar to that of *Mt*EgtB (Cα root mean square deviation is 0.77 Å using 397 atoms) (Fig. 4A,B).

The main structural differences are located in the surface loops and in regions corresponding to insertions/deletions, which adopt different conformations in *Mt*EgtB and *Pl*OvoA. Inspection of the active site regions of the two proteins reveals that, in analogy to what observed for *Mt*EgtB, the active site of *Pl*OvoA is located in a cleft at the bottom of a wide tunnel (Fig. 4C,D). The three conserved histidine residues His74, His169 and His173 (His51, His134 and His138 in *Mt*EtgB) coordinate the catalytic Fe ion; the Tyr residue in position 442 (Tyr377 in *Mt*EtgB) is in close proximity to the metallic center. The putative residues involved in the binding of *Mt*EgtB^30^ with its two substrates N-alpha-trimethyl histidine and gamma-glutamyl cysteine (i.e. Arg87, Arg90, Tyr380, Trp415, Asp416, Arg420) are not conserved in the structure of *Pl*OvoA, and this probably accounts for the different products of the reactions catalyzed by the two enzymes. Interestingly, other residues as Met108, Tyr442, Phe445, His480 and Phe481 are easily recognized in the structural model of the *Pl*OvoA active site (Fig. 4D), and are conserved in all marine metazoans OvoAs (see alignments in Fig. 3 and Supplementary Fig. 1).

### Evolutionary analysis of OvoA in metazoans

**Figure 5:**
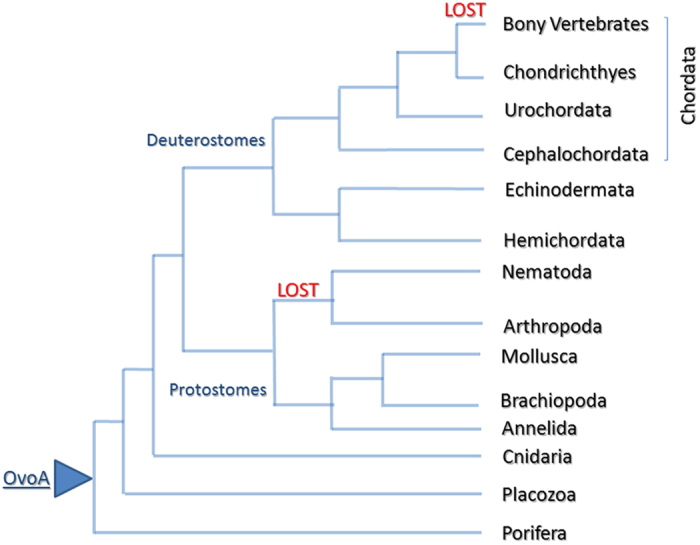
Schematic phylogenetic tree of OvoA in metazoans. The diagram is representative of the different metazoan phyla, in which OvoA is present (see Table 1 for protein sequence ID).

The evolutionary history of OvoA was surveyed in metazoans. We found OvoA with a high degree of conservation in porifera, placozoa, cnidaria (anthozoa), protostomes (annelida and mollusca) and deuterostomes. Among protostomes, *OvoA* gene was lost in nematoda (*Caenorhabditis elegans*) and arthropoda (*Drosophila melanogaster*), at least according to the genomic and transcriptomic data available at date. Among deuterostomes, we found OvoA in ambulacraria, i.e. echinodermata (*P. lividus* and *S. purpuratus)* and hemichordata (*Saccoglossus kowalevskii*), and in the chordate phylum in cephalochordata (*Branchiostoma floridae*), urochordata (*Ciona intestinalis*), and in chondrichthyes (*Callorhinchus milii*). Interestingly, in *S. kowalevskii* we found two genes coding for OvoA of 813 aa and 795 aa, respectively. The two proteins share 59% sequence identity. However, we could not identify OvoA orthologous in bony vertebrates, neither in Actinopterygii nor Sarcopterygii (Fig. 5 and Table 1), suggesting that a second independent event of gene loss took place in the ancestor of Osteichthyes fishes.

## Discussion

In this work, we investigated functional and evolutionary aspects related to the gene and the enzyme responsible for the biosynthesis of ovothiol, the methylated thiohistidine, first isolated in sea urchin eggs^5^. Although in the past decades ovothiol has been suggested to be involved in different biological processes^14^,^15^,^18^,^19^, its functions still remain a matter of debate. Here, combining biochemical and molecular studies with bioinformatics tools for available genomes screening, we provide new insights into the role of ovothiol in metazoans.

Since the eighties it has been suggested that ovothiol mainly acted to protect sea urchin eggs from the high oxidative burst at fertilization. Here, we demonstrate that ovothiol plays a role also during development. Indeed, our data showed that, after consumption of the *PlOvoA* maternal transcript, the levels of *PlOvoA* mRNA significantly increased at the pluteus stage. This profile overlapped that of ovothiol content in *P. lividus* early development^10^,^33^, suggesting a functional role for ovothiol also in the embryo. The *PlOvoA* gene expression regulated by stress factors, together with *in silico* analysis of the gene upstream region, demonstrated that in sea urchins *OvoA* acts as a metal and general stress responsive gene during development. The up-regulation of *PlOvoA* at the swimming blastula stage after exposure to 1 and 5 μM Cd^2+^ indicated that, under these conditions, the gene was regulated by the metal-induced stress, as suggested by the presence of MREs in the promoter region. At the prism stage, no significant regulation of gene expression was observed. However, the significant increase in ovothiol levels at this stage, suggested that, following *PlOvoA* mRNA increase at the swimming blastula stage, the synthesis of the metabolite was induced at the later prism stage. Finally, at the pluteus stage, *PlOvoA* was strongly down-regulated, indicating that the production of high amounts of ovothiol, at 5 μM Cd^2+^, may exert a negative feedback regulation on its synthesis, as already reported for other metabolic enzymes, including the one catalyzing the first step of glutathione biosynthesis^34^. At higher Cd^2+^ concentrations (10 μM) a negative regulation of *PlOvoA* occurred probably related to the marked toxicity of the metal. The increase in ovothiol content after Cd^2+^ treatment closely resembled the glutathione formation observed in marine organisms in response to metal and natural toxins exposure^35^,^36^,^37^,^38^ to counteract the increasing production of ROS. This indicates that ovothiol behaves similarly to the universal cellular antioxidant glutathione. Accordingly, the slight regulation of *PlOvoA* following Mn^2+^ treatment may be due to the essential biological role of the metal, which does not cause an increase of ROS levels in sea urchin embryos^39^. *PlOvoA* metal-regulation may be mediated by the highly homologous metal responsive transcription factor-1 (MTF-1), which accumulates in the nucleus upon heavy metal exposure. MTF-1 binding to the core consensus TGCRCNC sequence in MRE induces the expression of metallothioneins and other genes involved in metal homeostasis^40^.

As a consequence of metal-dependent ROS formation, Cd^2+^ can also modulate antioxidant gene transcription through SREs^38^. However, these elements usually mediate general stress responsive gene activation, including toxin-mediated stress. Our findings indicate that when sea urchins were cyclically exposed to toxic *O.* cf. *ovata* bloom, *PlOvoA* gene was strongly up-regulated during early development of the offspring, thus suggesting a general stress activation of *PlOvoA*. Interestingly, the promoter region of *PlOvoA* gene contains, besides SRE, also AP-1 and CRE sequences, mainly involved in the antioxidant response. The coexistence of these 3 regulatory elements further supported the view that *PlOvoA* is a stress and antioxidant responsive gene^41^.

Previous studies on the characterization of OvoA focused mainly on the kinetics and substrate specificities of the enzyme isolated from some microorganisms^26^. In this work, we identified the intron-exon composition of the gene and the protein primary structure of OvoA in sea urchins, as the first example in metazoans. The active site structural model of *Pl*OvoA in comparison with that of *Mt*EgtB allowed us to identify putative binding residues for the two specific substrates of OvoA, cysteine and histidine. These residues were highly conserved in OvoA from the other metazoans, suggesting their direct involvement in the catalysis of ovothiol production, instead of ergothioneine. These amino acidic substitutions can be considered particularly relevant to the evolutionary divergence of EgtB and OvoA, which likely accounts for adaption to different functional niches for the two enzymes.

**Figure 6:**
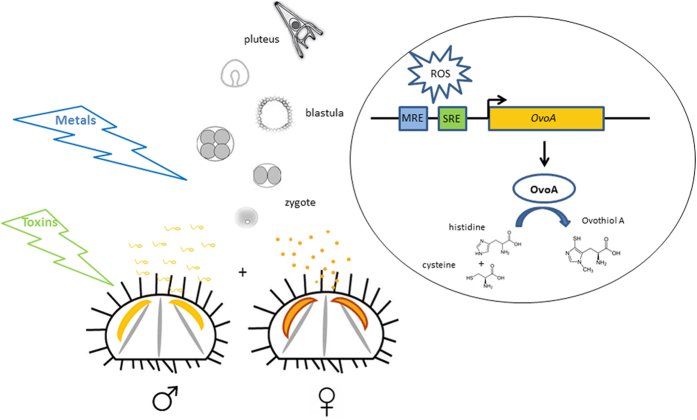
Proposed model for ovothiol biosynthesis regulation. Broadcast spawners, i.e. sea urchins, release eggs and sperm in the column seawater, where fertilization occurs. Toxins and heavy metals, dispersed in seawater, induce oxidative stress in the embryos. ROS production can affect transcription factors, which bind to MRE and/or SRE and activate *OvoA* transcription. Finally, OvoA enzyme catalyzes the synthesis of ovothiol to counteract redox unbalance.

Concerning the evolutionary history of OvoA in metazoans, the two independent gene loss events, one in nematodes and arthropods, at least in terrestrial species, and the other one in the ancestor of osteichthyes fishes suggested a crucial function of ovothiol biosynthesis in marine environment. Unfortunately, the lack of available genomes from marine nematodes and arthropods, did not allow to rule out the presence of OvoA in these invertebrates. The occurrence of OvoA in most of broadcast spawners (Table 1), suggested that this antioxidant gene protects the eggs and the embryos released in seawater from special external factors responsible for ROS production in marine organisms. We propose that invertebrate broadcast spawners (i.e. sea urchins) release gametes in the seawater column, where eggs and embryos are directly exposed to soluble natural toxins or heavy metals. These factors can induce oxidative stress in the embryos, thus activating transcription factors, which bind to MRE and/or SRE to promote *OvoA* transcription. Finally, OvoA enzyme catalyzes the synthesis of ovothiol to counteract redox unbalance (Fig. 6). However, the occurrence of OvoA also in invertebrates with internal fertilization, e.g. some cephalopods or some vertebrates, such as cartilaginous fishes, which can be oviparous or ovoviviparous (the embryos develop within the mother’s body), suggests a broader function for the enzyme. OvoA may play a key role in maintaining cellular redox homeostasis, i.e. in response to the different oxygen pressures. Indeed, in the early stages of development, embryo respiration occurs through a direct exchange of oxygen with the surrounding environment, whereas in the later stages, gas exchanges are guaranteed by fetal membranes (yolk sac, allantoid and placenta), usually highly vascularized in fishes. Therefore, we hypothesize that the loss of OvoA in bony fishes may be related to the appearance and development of the swim bladder and of more efficient systems of respiration and blood circulation, which allow a more controlled oxygen exchange with respect to the cartilaginous fishes and other invertebrates, which have conserved OvoA. Interestingly, the swim bladder, responsible for gas volume regulation and pressure, has a common origin with the lung in dipnoi and tetrapods. Moreover, among bony fishes, coelacanths and lungfishes are considered more closely related to tetrapods^42^ than to teleost fishes and represent key species to elucidate the transition from ancestral aquatic vertebrates to terrestrial animals^43^. The transition from the aquatic-to-aerobic environment requires accumulation of pre-adaptive changes in the genomes of ancestral species to adapt to an air-based environment, such as lung development and adaptation to a different oxygen pressure, as well as loss of genes no more necessary to the new conditions. Therefore, OvoA could represent one of the genes lost during the water to land transition, for the concomitance emergence of other mechanisms of oxygen pressure regulation and exchange. In agreement with this hypothesis, the presence in *S. kowalevskii* of two genes coding for OvoA may suggest a double specialization for the OvoA to adapt to different conditions of oxygen pressure. Indeed, the hemichordate lives in burrows on the sea-bed in anaerobic conditions and during low tide can be exposed to aerobic ones^44^.

In conclusion, this study demonstrates for the first time that *OvoA* can be regarded as a metal and a general stress responsive gene, and ovothiol a new biomarker of stress conditions in marine organisms. The OvoA evolutionary history in metazoans suggests the involvement of ovothiol in regulating redox homeostasis in organisms that have to survive in a marine habitat, where oxygen pressure and solubility is far from being similar to air-based environment.

## Methods

### Ethics Statement

*Paracentrotus lividus* (Lamarck) sea urchins were collected in the Gulf of Naples, near Castel dell’Ovo (Lat. 40°49′,69698, Lon. 14°14′,81328) from a location that is not privately-owned or protected in any way, according to the authorization of Marina Mercantile (DPR 1639/68, 09/19/1980 confirmed by D. Lgs. 9/01/2012 n. 4). The animals were also collected at the Marine Protected Area Gaiola at station G1 (40°47.494′N, 14°11.282′E) in October of 2013, in the framework of the “Ostreopsis Monitoring Plan” for the Campania Region (Italy). The field studies did not involve endangered or protected species. All animal procedures were in compliance with the guidelines of the European Union (directive 2010/63 and following D. Lgs. 4/03/2014 n. 26).

### Gamete collection

Sea urchins were collected during the breeding season by SCUBA divers in the Gulf of Naples, transported in an insulated box to the laboratory within 1 h after collection, and maintained in tanks with circulating seawater. To perform metal experiments, animals were acclimated for a minimum of 10 days until use. Fertilization was carried out as described in Migliaccio and collaborators (2014)^32^. Animals collected at Gaiola for the *in situ* study were fertilized with sperm from a pool of males collected in a control area, not affected by toxic microalgae blooms.

### Embryo culture, treatments and morphological analysis

Embryos (150 eggs/ml) were allowed to develop at 18 ± 2 °C in a controlled temperature chamber at 12:12 light:dark cycle. Metal treatment was performed 5 min from fertilization, by adding Cd^2+^ or Mn^2+^ ions under careful agitation. Nominal concentrations were 1, 5 and 10 μM for Cd^2+^ (cadmium chloride-Sigma-Aldrich) and 36 μM for Mn^2+^ (manganese chloride tetrahydrate-Sigma-Aldrich). Embryos derived from fertilization of females harvested at Gaiola were reared in the field (sampling) water. Experiments were performed in triplicate using the eggs collected from three different females. The development was followed by inverted microscope and the morphological observations were performed approximately 48 h post fertilization, on plutei collected and fixed in 4% formalin.

### RNA extraction and cDNA synthesis

Samples of fertilized eggs (about 1500) treated as described above were collected at each developmental stage by centrifugation at 1800 rcf for 10 min in a swing out rotor at 4 °C. The pellet was washed with phosphate buffered saline and then frozen in liquid nitrogen and kept at −80 °C. Total RNA was extracted from each developmental stage using RNAqueous- Microkit (Ambion) according to the manufacturer’s instructions. The amount of total RNA extracted was estimated by the absorbance at 260 nm and the purity by 260/280 and 260/230 nm ratios, by Nanodrop (ND-1000 UV-Vis Spectrophotometer; NanoDrop Technologies). The integrity of RNA was evaluated by agarose gel electrophoresis. Intact rRNA subunits (28S and 18S) were observed on the gel indicating no degradation of the RNA. For each sample, 600 ng of total RNA extracted was retro-transcribed with iScript^TM^ cDNA Synthesis kit (Biorad), following the manufacturer’s instructions. cDNA was diluted 1:5 with H_2_O prior to use in Real Time qPCR experiments.

### Gene expression by Real Time qPCR

For real time qPCR experiments the data from each cDNA sample were normalized using the gene encoding for the zinc-finger transcription factor *Pl-Z12-1* as well-assessed endogenous control during *P. lividus* development^32^,^45^. For *Pl-Z12-1*, we used primers reported in the previous paper (32). In the case of OvoA, specific primers were designed on the basis of nucleotide sequence using Primer 3: forward primer 5′-AGGTCAGCATGGACATAGCC-3′, reverse primer: 5′-CCTCAGCCGACTTCAAGAAC-3′. The amplified fragment (156 bp in length) using Taq High Fidelity PCR System (Roche) was purified from agarose gel using QIAquick Gel extraction kit (Qiagen) and specificity of PCR product was checked by DNA sequencing. Specificity of the amplification reaction was verified by melting curve analysis. The efficiency of primer pair was calculated according to standard methods curves using the equation E = 10^−1/slope^. Five serial dilutions were set up to determine Ct values and reaction efficiencies for the primer pair. Standard curve was generated for oligonucleotide pair using the Ct values versus the logarithm of dilution factor. PCR efficiencies were calculated for control and target gene and were found to be about 2. Diluted cDNA was used as a template in a reaction containing a final concentration of 0.3 μM for each primer and 1x FastStart SYBR Green master mix (total volume of 10 μl). PCR amplifications were performed in a ViiA^TM^ 7 Real Time PCR System (Applied Biosystems) thermal cycler using the following thermal profile: 95 °C for 10 min, one cycle for cDNA denaturation; 95 °C for 15 sec and 60 °C for 1 min, 40 cycles for amplification; 72 °C for 5 min, one cycle for final elongation; one cycle for melting curve analysis (from 60 °C to 95 °C) to verify the presence of a single product. Each assay included a no-template control for each primer pair. To capture intra-assay variability all Real Time qPCR reactions were carried out in triplicate. Fluorescence was measured using ViiA^TM^ 7 Software (Applied Biosystems). The expression of OvoA gene was analyzed and internally normalized against *Pl-Z12-1* using Relative Expression Software Tool software (REST) based on Pfaffl method (2002)^46^. Relative expression ratios above two cycles were considered significant.

### Determination of ovothiol A disulphide concentration in sea urchin embryos

Embryos, harvested at different developmental stages were homogenized in ethanol-1 M HCl 80:20 v/v (1 mL) and left overnight at room temperature under stirring in the air. After centrifugation at 14000 g for 15 min at 4 °C, the supernatant was recovered. The pellet was washed three times with acidic ethanol and the combined supernatants, concentrated to a small volume, were extracted three times with the same volume of ethyl ether freed from peroxide by passage over alumina column. The aqueous layer was concentrated to small volume and loaded onto a Dowex 50WX2, column (1 cm × 2 cm). Elution was sequentially carried out with water, 0.1 M and 0.5 M HCl. The column was then eluted with 4 M HCl and the collected fractions were monitored spectrophotometrically in the 200–350 nm range. Fractions exhibiting the UV spectrum typical of ovothiol were collected, concentrated to a volume of 100 μL for HPLC analysis on a column Phenomenex Synergi Sphereclone (25 cm × 0.46 cm, 5 *µ*m particle size) with an isocratic elution in 1% formic acid taken to pH 4.5 with ammonia.

### Statistical analysis

Data are presented as means ± SEM and analyzed by One-way ANOVA (P < 0.05) with Turkey’s Multiple Comparison Test and Two-way ANOVA (P < 0.05), with Bonferroni post hoc test as reported in figure legends. Statistical analysis was performed using GraphPad Prism version 4.00 for Windows (GraphPad Software, San Diego California USA). The results of biochemical experiments were reported as means ± SEM and analyzed by unpaired t-test for comparison between the groups. P < 0.05 was considered statistically significant. For Real Time qPCR analysis, results were reported as means ± SEM and significance was tested using the “Pair Wise Fixed Reallocation Randomisation Test”, developed by REST software^47^. The number of experiments was reported in the figure legends.

### Bioinformatic analysis

OvoA genomic sequences or transcripts were downloaded from the following sources: *S. purpuratus* from Spbase (www.spbase.org), *P. lividus* from BioDev (www.octopus.obs-vlfr.fr), *Ciona intestinalis* and other invertebrate species from Ensemble (www.ensemble.org), *Callorhinchus milii* from the Elephant shark Genome Project (www.sharkgenome.imcb.a-star.edu.sg), *Platynereis dumerilii* was from http://jekely-lab.tuebingen.mpg.de/blast/48. Amino acid sequence of OvoA from different sources was found by tblastx and Blastp homology search using as template *Sp*OvoA gene sequence. OvoA protein sequence alignments were obtained by ClustalW^49^. 12 kb upstream of *S. purpuratus* and *P. lividus OvoA* genes were scanned for searching the following metal responsive DNA cis-elements: Metal Response Element [MRE consensus TGC(A/G)CNC]^50^, Stress Response Element [SRE consensus CCCCT], cAMP-Responsive Element [CRE consensus T(G/T)ACGT(A/C)A], and AP-1 [TTACTAA]^41^.

### Structural modelling

The model of *Pl*OvoA structure was obtained using the structure of the Ergothioneine biosynthesis enzyme EgtB from *Mycobacterium thermoresistibile* as starting model (*Mt*EtgB, PDB code 4 × 8B^30^, and the SWISS-MODEL server (http://swissmodel.expasy.org/). The program DeepView-Swiss-PdbViewer^51^ was used to include iron and iron-coordinated water molecules close to the conserved His residues in the active site. The position of the conserved His residues was assumed to be similar to that of *Mt*EgtB. DeepView-Swiss-PdbViewer was also used to minimize the energy of the structure and calculate the root mean square deviation from the starting model. The figures were done with Pymol (www.pymol.org).

## Additional Information

**How to cite this article**: Castellano, I. *et al.* Shedding light on ovothiol biosynthesis in marine metazoans. *Sci. Rep.* **6**, 21506; doi: 10.1038/srep21506 (2016).

## Supplementary Material

Supplementary Information

## Figures and Tables

**Table 1:** OvoA in metazoans.

Species	Phylum	Class	OvoA ID	Ovothiol	Reproduction	Reproductive behaviour	Habitat
*Trichoplax adhaerens*	Placozoa		XP_002114341	n. d.	generally asexual		marine
*Amphimedon queenslandica*	Porifera	demospongiae	XP_003388095	n. d.	sperm spawner		marine
*Nematostella vectensis*	Cnidaria	anthozoa	XP_001630288	n. d.	asexual or sexual		marine
*PROTOSTOMES*							
*Capitella teleta*	Annelida	polychaeta	ELU18927	n. d.	external	oviparous	marine
*Platynereis dumerilii*	Annelida	polychaeta	lcl|N4464	yes	spawner	oviparous	marine
*Lottia gigantea*	Mollusca	gastropoda	XP_009056382	n. d.	spawner	oviparous	marine
*Crassostrea gigas*	Mollusca	bivalvia	EKC24550	n. d.	spawner	oviparous	marine
*Aplysia californica*	Mollusca	gastropoda	XP_005099570.1	n. d.	internal	oviparous	marine
*Biomphalaria glabrata*	Mollusca	gastropoda	XP_013083905.1	n. d.	internal	oviparous	freshwater
*Octopus bimaculoides*	Mollusca	cephalopoda	KOF75235	n. d.	internal	oviparous	marine
*Octopus vulgaris*	Mollusca	cephalopoda	*	yes	internal	oviparous	marine
*Lingula anatina*	Brachiopoda	lingulata	XP_013417309.1	n.d.	spawner	oviparous	marine
*Caenorhabditis elegans*	Nematoda	chromadorea	–	n.d.	internal		terrestrial
*Drosophila melanogaster*	Arthropoda	insecta	–	n.d.	internal	oviparous	terrestrial
*DEUTEROSTOMES*							
*Dermasterias imbricate*	Echinodermata	Asteroidea	n. a.	yes	egg spawner	oviparous	marine
*Paracentrotus lividus*	Echinodermata	Echinoidea	**	yes	egg spawner	oviparous	marine
*Strongylocentrotus purpuratus*	Echinodermata	Echinoidea	XP_789318.2	yes	egg spawner	oviparous	marine
*Saccoglossus kowalevskii*	Hemichordata	Enteropneusta	XP_002741469	n.d.	external	oviparous	marine
*Saccoglossus kowalevskii*			XP_006824967				
*Ciona intestinalis*	Chordata	Ascidiacea	XP_009858657	n.d.	egg spawner	oviparous	marine
*Branchiostoma floridae*	Chordata	Leptocardii	XP_002602505	n.d.	egg spawner	oviparous	marine
*Callorhinchus milii*	Chordata	Chondrichthyes	XP_007907153	n.d.	external	oviparous	marine
*Gadus morhua*	Chordata	Actinopterygii	–	n.d.	external	oviparous	marine
*Takifugu rubipres*	Chordata	Actinopterygii	–	n.d.	external	oviparous	marine
*Gasterosteus aculeatus*	Chordata	Actinopterygii	–	n.d.	external	oviparous	marine
*Salmo salar*	Chordata	Actinopterygii	–	n.d.	external	oviparous	marine
*Latimeria chalumnae*	Chordata	Sarcopterygii	–	n.d.	internal	ovoviviparous	marine
*Xenopus tropicalis*	Chordata	Amphibia	–	n.d.	internal	oviparous	terrestrial
*Gallus gallus*	Chordata	Aves	–	n.d.	internal	oviparous	terrestrial
*Loxodonta africana*	Chordata	Mammalia	–	n.d.	internal	viviparous	terrestrial
*Homo sapiens*	Chordata	Mammalia	–	n.d.	internal	viviparous	terrestrial
*Mus musculus*	Chordata	Mammalia	–	n.d.	internal	viviparous	terrestrial

Occurrence of OvoA and ovothiol, reproductive strategy and habitat in different metazoan species.

^*^Dr. Graziano Fiorito personal communication.

^**^This work identification; n.a. not available genome; n.d. not determined.
