# Supplementary material for: Shedding light on ovothiol biosynthesis in marine metazoans
Source: Sci Rep. 2016 Feb 26;6:21506. doi: 10.1038/srep21506 (PMC4768315; doi:10.1038/srep21506)
Supplement: Supplementary Information [file srep21506-s1.doc]

Shedding light on ovothiol biosynthesis in marine metazoans

Immacolata Castellano, Oriana Migliaccio, Salvatore D’Aniello, Antonello Merlino, Alessandra Napolitano, Anna Palumbo

Supplementary Figure S1. Aminoacid alignment of OvoA from metazoans. The conserved residues likely accounting for binding to cysteine and histidine (Met108, Tyr442, Phe445, His480 and Phe481 in PlOvoA) are boxed in yellow and the putative iron-binding motif is boxed in red.

S.purpuratus EMSWDDTENYRMGG-SYQWPSLENVVQFRLKVRNVIRKVIEDTPLELPVTQES-------

P.lividus EMSWDDTENYRMGG-SYQWPSLKDVVQFRLKVRNVIRKVIEDTPLELPVTQES-------

S.kowalevskii EMSWDDTENYRMGG-SFKWPTLTEVTMYRRKVRQMVLKTIQDTPLVLPIHQES-------

S.kowalevskii2 EMSWDDTENYRMGG-TFKWPSIEEVTVYRRTVRNLILKVIADTPLVLPINQES-------

A.queenslandica EMSWDDTENYRMGG-SYQWPSLSEVMEYRSRVRELVIEVIENAPLTLPITMDH-------

B.floridae EMSWDDTENYRMGG-RFQWPPLAEAVEFRRLVRNRIRQLIEDTPLQLPITQDNPWATPTK

T.adhaerens EMSWDDTENYRMGG-NFKWPSVKDILDYRAKVRKVVLDVIDTAPLVLPITQDS-------

C.intestinalis EMSWDDTENYRMGG-AYKWPPIEKITEYRKKVREIVRSVIQNLDLTFPVTEDS-------

A.californica EMSWDDTENYRMGG-SYKWPAISEVVEYRQIVRDLILKVIDDTPLELPVTMDS-------

B.glabrata EMSWDDTENYRMGG-SYKWPALHEVVEYRRTVRSIILKVIESTKLELPINMES-------

C.gigas EMSWDDTENYRMGG-SYKWPSLSSVVEYRRNVRNLVLKVIHDTPLDLPVTMES-------

L.gigantea EMSWDDTENYRMGG-SYKWPTIESVVDYRRQVRNIITNIIEKTPLELPINMES-------

C.teleta EMSWDDTENFRMGG-QLKWPKLDNVVDYRRKVRDVIINIIKKTPLQLPINMDS-------

P.dumerilii EMSWDDTENYRMGG-KVSWPWVKRVADYRQKVKELILKVIEETPLELPVTHDS-------

L.anatina EMSWDDTENFRMGG-QYKWPNLKDVVEYRRQVREVILQVIENTPLELPITKES-------

N.vectensis EMSWDDTENFRMGG-EFTWPKVSAVAEYRRRVREVILEIIETTPMELPITQGS-------

C.milii EMFWDNTESSRMGG-RYHWPELQECVEYRRRVRLLIREVIRQTPLTLPITTNS-------

O.bimaculoides EMFWDDTVNQRIDGKPIVWPEVVDVTAYRRQVKNAILQMINDTPLELPITMES-------

** **:* . *:.* ** : :* *: : . * : :*:

S.purpuratus ---------------------------KWWALFMGFEHERIHIETSSVLIRQLPVSLVRK

P.lividus --------------------------KWWAMFMGFEHERIHIETSSVLIRQLPIGMVKT

S.kowalevskii ---------------------------EWWALIMGIEHERIHIETSSVLIRQLPVEFVQI

S.kowalevskii2 ---------------------------PWWALLMGIEHERIHIETSSVLIRQLPVEFVQI

A.queenslandica ---------------------------PWWSIFMGLEHERIHIETSSVLIRQLPVDYLEC

B.floridae VRSAGWLPWLPEQNSLAISLATRSCLAPRWAVMMGVEHEKIHIETSSVLIRQMPITMVTT

T.adhaerens ---------------------------PWWAVMLGLDHERIHFETSSVLIRQMPVDLVTK

C.intestinalis ---------------------------PCWSVWLAIEHERIHLETSSVLIRQMPIDMVSR

A.californica ---------------------------PWWALMMGMEHERIHLETSSVLIRQMPIEMVQR

B.glabrata ---------------------------PWWALLMGMEHERIHLETSSVLIRQLPIEIVVR

C.gigas ---------------------------PWWAILMGIEHERIHLETSSVLIRQLPVEMVTK

L.gigantea ---------------------------PWWALVMGMEHERIHLETSAVLIRQLPVDMVTK

C.teleta ---------------------------PWWGLFMGMDHERIHIETSSVLIRQLPVELVEC

P.dumerilii ---------------------------PWWSLFMGMEHERIHLETSSVLIRQLPISMVTN

L.anatina ---------------------------PWWSLFMGMEHERIHHETSSVLIRQLPVDMVTR

N.vectensis ---------------------------PWWALFMGMEHERIHLETSSVLIRQLPLSYVQR

C.milii ---------------------------IWWPLLMGFEHERIHLETSSVLVRQMPVHLVRK

O.bimaculoides ---------------------------KWWALLMGMAHERIHLETSSVLIRQLPVNVVTK

* : :.. **:** ***:**:**:*: :

S.purpuratus NETHHLYDDFSSPTYDGHHNIILGGSWMSTGDEASKFARYAFRRHFFQHAGFRLARSASP

P.lividus SETHYLYDDFSSPTYDGKHNVILGGSWISTGDEASRFARYAFRRHFFQHAGFRLARSCS-

S.kowalevskii TETSFLYDDFSAPLHDGKHNMMLGGSWITNGTTASKYCRTGYRRHFFQHAGFRLARSVTT

S.kowalevskii2 NKTHFLYDDFSSPCFDGNHTMMQGGSWVSTGGATSRYARFSFRRHFYQHLGFRLAKSVNV

A.queenslandica YESHYLYDDFSSPCFDGRHNMIQGGSWASTGDLCSRFARYAFRRHFYQHMGFRLVRSLSP

B.floridae YRQHHLYDDYAFPFCDGRHGLMLGGSWASNGTYNSRFSRSFFRRHFYQHAGFRVARTLPG

T.adhaerens FKSHYVYHDFSTPCFDGRHNMIMYGSWASTGGLSSCYARFAFRRHFFQHMGFRVVRHNPD

C.intestinalis YTLNKYYHDYSAPGLDGSHGVIVGGSWISTGNEASRFSRYGFRRHFMQHAGFRVARTLDG

A.californica FHTHFLYDDFSSPCFDGKHNIILGGSWISTGDEASRFARYAFRRHFFQHCGFRIARSL-T

B.glabrata FHSHFLYDDFSSPCFDGKHNVILGGSWISTGDEASRFARYAFRRHFFQHCGFRLAR----

C.gigas YSSHWYYDDFSSPCFDGRHNLILGGSWVSTGDEASKFARFAFRRHFIQHAGFRIARS--L

L.gigantea FKSHHFYDDFSTPTFDGKHHLILGGSWISTGNEASRFARYAFRPHFVQFAGFRTVRN---

C.teleta FQTSPLYDDFSSPCFDGRHNLILGGSWISSASAASRFARYAFRRHFFQHMGFRLARSLHH

P.dumerilii YDTHHYYDDFSSPTYDGRHNVIMGGSWISTGDEASRFARYAFRRHFFQHCGFRVVRNIPS

L.anatina QDTHWLYDDFSTPCFDGRHNMIMGGSWITTGDSASRFARFSFRRHFFQHAGFRLVESIGP

N.vectensis NDTHYLYDDFSSPTFDGKHYMIMGGSWVSTGDEASRFARFSFRPHFFQHLGFRLVRSATA

C.milii FDTVYLYDDFSAPCFDSQHTVILGGSWCSTGDEASRFARFAFRRHFFQHLGFRLAKSR--

O.bimaculoides -----------------------NGSWISTGNEASRFARYAFRRHFFQHLGFRMVKSSHD

*** :.. * :.* :* ** *. *** ..
